# Human Anti-CCR4 Minibody Gene Transfer for the Treatment of Cutaneous T-Cell Lymphoma

**DOI:** 10.1371/journal.pone.0044455

**Published:** 2012-09-04

**Authors:** Thomas Han, Ussama M. Abdel-Motal, De-Kuan Chang, Jianhua Sui, Asli Muvaffak, James Campbell, Quan Zhu, Thomas S. Kupper, Wayne A. Marasco

**Affiliations:** 1 Department of Cancer Immunology and AIDS, Dana-Farber Cancer Institute, Harvard Medical School, Boston, Massachusetts, United States of America; 2 Department of Medicine, Harvard Medical School, Boston, Massachusetts, United States of America; 3 Department of Dermatology, Brigham and Women’s Hospital, Harvard Skin Disease Research Center, Harvard Medical School, Boston, Massachusetts, United States of America; Federal University of São Paulo, Brazil

## Abstract

**Background:**

Although several therapeutic options have become available for patients with Cutaneous T-cell Lymphoma (CTCL), no therapy has been curative. Recent studies have demonstrated that CTCL cells overexpress the CC chemokine receptor 4 (CCR4).

**Methodology/Principal Findings:**

In this study, a xenograft model of CTCL was established and a recombinant adeno-associated viral serotype 8 (AAV8) vector expressing a humanized single-chain variable fragment (scFv)-Fc fusion (scFvFc or “minibody”) of anti-CCR4 monoclonal antibody (mAb) h1567 was evaluated for curative treatment. Human CCR4^+^ tumor-bearing mice treated once with intravenous infusion of AAV8 virions encoding the h1567 (AAV8-h1567) minibody showed anti-tumor activity *in vivo* and increased survival. The AAV8-h1567 minibody notably increased the number of tumor-infiltrating Ly-6G^+^ FcγRIIIa(CD16A)^+^ murine neutrophils in the tumor xenografts over that of AAV8-control minibody treated mice. Furthermore, in CCR4^+^ tumor-bearing mice co-treated with AAV8-h1567 minibody and infused with human peripheral blood mononuclear cells (PBMCs), marked tumor infiltration of human CD16A^+^ CD56^+^ NK cells was observed. The h1567 minibody also induced *in vitro* ADCC activity through both mouse neutrophils and human NK cells.

**Conclusions/Significance:**

Overall, our data demonstrate that the *in vivo* anti-tumor activity of h1567 minibody is mediated, at least in part, through CD16A^+^ immune effector cell ADCC mechanisms. These data further demonstrate the utility of the AAV-minibody gene transfer system in the rapid evaluation of candidate anti-tumor mAbs and the potency of h1567 as a potential novel therapy for CTCL.

## Introduction

Cutaneous T cell lymphomas (CTCLs) are a clinically heterogeneous group of lymphoproliferative malignancies characterized by the clonal accumulation of mature and skin-homing memory T cells. Mycosis fungoides (MF), which is the most common and indolent form of CTCL, accounts for 50%–60% of CTCL cases [1]; primary cutaneous CD30^+^ lymphoproliferative disorders, more specifically primary cutaneous anaplastic large cell lymphoma (PC-ALCL) – the second most common CTCL, accounts for circa 30%; and Sézary syndrome, which is an aggressive leukemic variant of CTCL, affects approximately 5% of patients. These patients exhibit significant immune dysfunction [2], [3] because of the global dysregulation of T cells, which is due to an unknown etiology [4], [5]. Bacterial sepsis is the terminal event in most patients with advanced cancer. Current therapies for patients with advanced CTCL, including its leukemic variant, are only palliative, and extensive long-term remissions are rare. The poor 5-year survival rate of these patients receiving existing therapies clearly emphasizes the need for the development of new targeted therapies in this fatal disease [6].

Over the past few years, several studies have described the expression of chemokine receptors in the skin and blood of CTCL patients, including the uniformly high expression of CC chemokine receptor 4 (CCR4) [7], [8], [9], [10]. CCR4 is highly expressed in both leukemic CTCL including Sézary syndrome and in MF, both in the very early stages (patch and plaque stages) of the disease and in large cell transformations [7], [8], [10], [11], [12]. It is also expressed on circa 60% of PC-ATCL cells [1]. In a recently published consensus article regarding the classification of CTCL, it is clear that CCR4 is expressed in the vast majority of CTCL cells, regardless of their histological subtype [1]. On the other hand, expression of CCR4 is limited amongst non-malignant cells [13]. It is not present on neutrophils, monocytes, or B cells [14]. It is absent on naïve T cells, and present on fewer than half of all memory T cells [15].

While expression of CCR4 by tumor cells is associated with their skin involvement, CCR4 also has an important role in normal and tumor immunity [13], [14]. CCR4 is expressed at high levels on T regulatory cells (Tregs) that can migrate to tumor cells that secrete the CCR4 chemokines CCL17 and CCL21 to facilitate evasion from immune surveillance [16], [17]. High expression of the these CCR4 ligands has been detected in CTCL lesions [11], breast cancer [16], ovarian cancer [17] and oral squamous cell carcinoma [18]. Thus, targeted therapy against CCR4 may be an attractive treatment option for these malignancies, not only to directly kill the CCR4^+^ tumor cells, but also to overcome the suppressive effect of CCR4^+^ Tregs on the host anti-tumor immune response.

Monoclonal antibody (mAb)-based immunotherapies have become the standard therapy in an increasing number of human cancers [19], [20]. Tumor targeting with a human mAb directed against tumor-associated markers, such as CCR4, might provide a powerful therapeutic strategy against CTCL. In this study, we used recombinant adeno-associated viral (AAV) vector-mediated antibody gene transfer into SCID-BEIGE mice to evaluate the effectiveness of h1567, a novel humanized anti-CCR4 mAb to inhibit CCR4^+^ tumor cell growth and increase survival. The CCR4-specific antibody gene was packaged into an AAV vector and then delivered by a single direct intravenous (i.v.) injection which leads to the endogenous synthesis and durable expression of therapeutic antibody levels for months. Intravenous delivery of this h1567 minibody-encoding AAV vector allowed for rapid and accurate assessment of its therapeutic potential, thereby avoiding *ex vivo* manipulations involved in the production and purification of therapeutic mAbs.

*In vivo* studies using therapeutic mAb gene transfer after CCR4+ tumor cell implantation demonstrated the potent antitumor activity of the mAb h1567. In addition, the *in vivo* effector cells that mediate tumor cell killing through h1567 Fc binding to Fcγ receptors, namely FcγRIIIa (CD16A), were delineated. These studies suggest that mAb 1567 can serve as an effective antibody-directed therapy for immunodepleting malignant CTCL cells and may minimize collateral damage to the already compromised immune system. Furthermore, in the context of anti-cancer mAb therapies that require frequent and repeated administration, this AAV-based therapeutic antibody gene transfer strategy might serve as an alternative platform for their delivery.

## Results

### *In vitro* and *in vivo* Expression of AAV8-encoding Anti-CCR4 h1567

**Figure 1:**
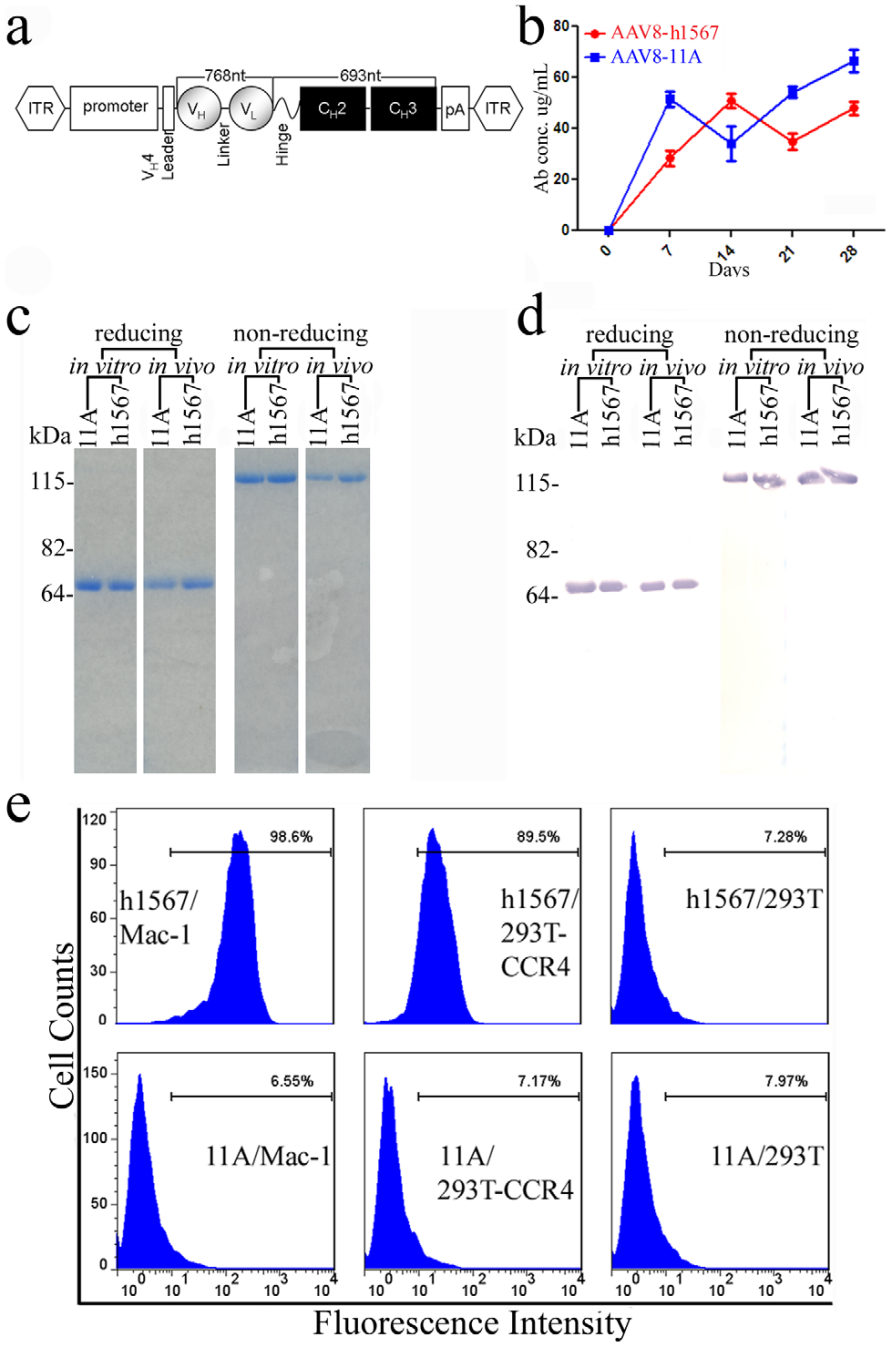
AAV vector construction and the expression of human mAbs in AAV-transduced mice. (**a**) Schematic representation of AAV-single chain variable region antibody (scFv) - human IgG1 Fc fusion (scFvFc) or “minibody” construct. Human mAb 11A (control) and “humanized” h1567 genes encoding the V domains of heavy (VH) and light (VL) chains were cloned between the AAV internal terminal repeats (ITRs) contained in vector pTRUF and expressed as a minibody protein. (**b**) *In vivo* transduction with AAV8-h1567-scFvFc and AAV8-11A-scFvFc in SCID-BEIGE mice after administering 2×10^11^ vg (viral genome) units per mouse by a single intravenous (i.v.) tail vein injection and in a final volume of 150 ul PBS. Serum levels were measured over time by human IgG ELISA. (**c**) SDS-PAGE confirming the molecular weight and disulfide-bond integrity of the 11A and h1567 minibodies. (**d**) Western blotting analysis of the monomer and dimer forms of the 11A and h1567 minibodies using an anti-human IgG1-Fc antibody and processed under reducing and non-reducing conditions. (c & d) minibody proteins recovered from *in vitro* culture (left) and serum following *in vivo* transduction (right) are shown. (**e**) Binding specificity of the AAV8-derived h1567 minibody. The specific binding of h1567 scFv-Fc in serum was shown using CCR4-positive cell lines, Mac-1 and 293T-CCR4 by flow cytometry. An equivalent concentration of the control 11A minibody did not show any binding. 293T cells serve as CCR4 negative control cells.

A modified scFvFc minibody format was used as the antibody moiety in the AAV8 vector, in which the V domains of heavy (VH) and light (VL) chains of the humanized scFv h1567 were fused to the coding region of the hinge and constant domains 2 and 3 (CH2 and CH3) of the human IgG1 heavy chain, to yield bivalent binding to the target molecule hCCR4 (**Figure 1a**) (DK. Chang et al., in press). The resulting recombinant AAV8 vector was used for both *in vitro* protein synthesis and virus production for *in vivo* antibody gene delivery. In a pilot dosing study, nude mice received a single injection of two different concentrations of AAV8-h1567 via intravenous tail vein injection. Serum h1567 minibody levels were followed for 15 weeks. H1567 minibody levels rose for the first 2–3 weeks, reaching levels of circa 65 and 96 ug/ml for the low (0.8×10^11^ vg/mouse) and high (2.0×10^11 ^vg/mouse) vector doses, respectively and then through the remaining weeks of the study leveled off at near peak levels for the high dosed vector and circa 1/3rd that level (∼35 ug/ml) for the low dosed vector (**Figure S1**). Because 2×10^11^ vg per mouse gave higher serum levels of h1567, this vector concentration was used in the subsequent *in vivo* studies.

CCR4^+^ Mac-1 tumor cells grow well in SCID-BEIGE mice and therefore we established a SCID-BEIGE/Mac-1 xenograft tumor model to evaluate the efficacy of AAV8-h1567 therapeutic minibody gene transfer. In SCID-BEIGE mice treated with a single intravenous tail vein injection of the AAV8 vectors, a time-dependent increase in serum concentrations of the control 11A and h1567 minibodies, reaching steady state levels of circa 50 ug/ml after 7–14 days and remaining at those peak levels through day 28, the last day of the study (**Figure 1b**). The control 11A is a irrelevant minibody that is directed against SARS Spike protein [21]. To determine whether the AAV8-minibody transduction *in vivo* could result in production of properly folded scFvFc, protein A-purified minibodies recovered from serum of SCID-BEIGE mice three weeks following intravenous delivery of AAV8 vectors were analyzed by sodium dodecyl sulfate polyacrylamide gel electrophoresis (SDS-PAGE) and Western blotting. As shown in **Figure 1c**, when examined under reducing conditions, the 11A and h1567 minibodies recovered from both *in vitro* and *in vivo* sources showed bands at the expected size for scFvFc, circa 60 kD. Analysis under non-reducing conditions showed dimer formation (mol wt circa 120 kD), thereby confirming that the minibodies were divalent *in vitro* and *in vivo* (**Figure 1c**). In addition, the ease of recovery of the AAV8-derived minibodies from serum using affinity purification on protein A, their reactivity on Western blot with the anti-human Fc antibody, and their stable dimer formation confirms the proper folding and structural integrity of their CH2-CH3 domains (**Figure 1c and 1d**).

### Binding Activity of h1567 Minibody in Serum Following AAV8-mediated Gene Transfer

To determine the functional integrity of the AAV8-derived scFvFc minibodies, sera obtained from mice 14 days after *in vivo* AAV8 transduction were examined for the level of binding to CCR4 by flow cytometry. As shown in **Figure 1e**, the secreted h1567 minibody in the mouse serum could specifically bind to the CCR4^+^ Mac-1 cells and CCR4^+^293T cells but not to parental 293T cells, indicating that the scFv domain was correctly folded and that it retained full antigen-binding activity. Irrelevant 11A minibody, which served as a negative control, did not bind to CCR4-expressing cells.

### Treatment of Pre-established Tumor-bearing Mice with AAV8-h1567

**Figure 2:**
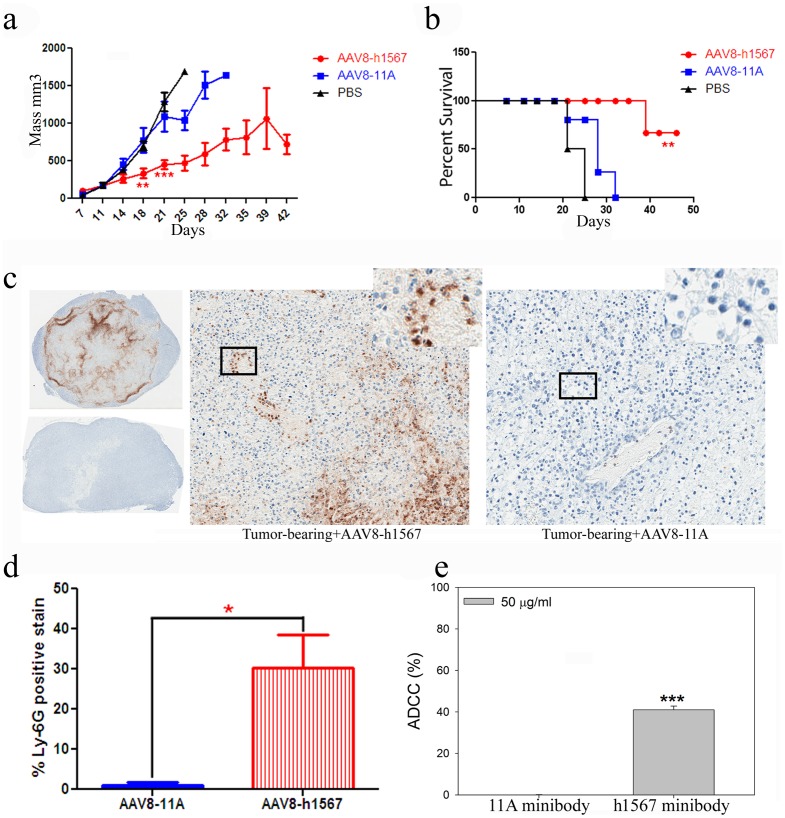
Anti-tumor effect of AAV8-derived h1567 minibody. (**a**) The tumor volume of each individual tumor plotted as a function of times (days post inoculation). AAV vectors were delivered intravenously by tail vein injection 7 days after the inoculation of 2.5×10^6^ Mac-1 tumor cells. **P<0.01, ***P<0.0005 when comparing tumor mass in AAV8-h1567-treated and control vector AAV8-11A-treated group on Day 18 and Day 21, respectively. (**b**) Survival analysis of AAV8-h1567 or control AAV8-11A-treated tumor-bearing mice (engrafted with 2.5×10^6^ Mac-1 tumor cells). Tumor-bearing PBS-treated mice were used as background controls. Statistically significant difference was observed between 1567 minibody-treated group and control groups (P<0.01). (**c**) Immunohistochemical analysis of representative tumor sections with anti-Ly-6G, a specific mAb recognizing murine neutrophils. The immunostaining shows tumor-infiltrating neutrophils (brown stain) in tumor from the SCID-BEIGE mice 21 days after administration of AAV8-h1567 encoding anti-CCR4 minibody (upper-left for entire tumor section and center-panel for magnified section). No staining was seen in the tumor from the mice treated with control vector AAV8-11A (lower-left and right panel). (**d**) Quantification of neutrophil infiltration from panel C. Entire tumor sections were captured using the Aperio ImageScope instrument, and the percentage of positively stained cells were quantitated by using a color deconvolution algorithm. *P<0.05. (**e**) *In vitro* ADCC activity against Mac-1 cells in the presence of h1567 minibody. The ADCC activity was assessed using purified SCID-BEIGE neutrophils as effector cells and CCR4+ Mac-1 cells as target cells. Neutrophil-mediated lysis of target cells was induced at an E:T ratio of 80∶1 in the presence of 50 ug/ml purified h1567 minobodies. The figure shown is representative of three independent experiments. *P<0.05, **P<0.01, ***P<0.0005. All data are represented as the mean ± SD.

The therapeutic effects of AAV8-h1567 gene transfer were next evaluated *in vivo* in SCID-BEIGE mice that carried subcutaneously implanted Mac-1 tumor xenografts. Groups of 4 mice were given a single intravenous injection of AAV8-h1567 or control AAV8-11A vector on day 7 after tumor inoculation and tumor volume was assessed twice weekly. As shown in **Figure 2a**, a single injection of AAV8-h1567 resulted in significantly reduced tumor growth compared with AAV8-11A treated mice or PBS control treated mice (*P*<0.01 at day 18, *P*<0.0005 at day 21). Mouse survival was monitored for up to 2 months. Tumor-bearing mice treated with AAV8-h1567 significantly outlived (*P*<0.005) mice treated with AAV8-11A or untreated mice (**Figure 2b**).

### Mechanisms of Tumor Cell Killing by h1567 in SCID-BEIGE Mice

Since SCID-BEIGE mice lack T and B lymphocytes as well as functional natural killer (NK) cells, it is possible that the CCR4^+^ Mac-1 tumor cells were eliminated by h1567 through neutrophil-dependent ADCC as neutrophils are intact in SCID-BEIGE mice and they express FcγRIIIA receptors which have been shown to mediated ADCC [22], [23]. Tumor sections were excised 21 days after AAV8 gene transfer and analyzed histologically for expression of Ly6G, a member of the Ly-6 family of glycosyl-phosphatidylinositol (GPI)-anchored proteins expressed on murine neutrophils [24], [25]. Immunostaining of tumors sections with neutrophil-specific Ly-6G mAb confirmed infiltration of neutrophils into tumors treated with AAV8-h1567 (**Figure 2c**, upper-left and middle panels) but not with AAV8-11A (**Figure 2c**, lower-left and right panels). Quantification of the neutrophil infiltration demonstrated a marked accumulation of Ly-6G+ staining cells only in the h1567 treated mice (**Figure 2d**).

To further assess the h1567-mediated, mouse neutrophil-dependent tumor cell killing, *in vitro* ADCC assay was carried out using purified SCID-BEIGE mouse neutrophils and h1567 minibody. Coculturing Mac-1 cells with mouse neutrophils in the presence of h1567 at the effector to target ratio of 80∶1 resulted in significant neutrophil-mediated ADCC as measured by lactate dehydrogenase (LDH) release from Mac-1 cell (**Figure 2e**). Control 11A minibody was not able to induce neutrophil-mediated cytotoxicity. These *in vitro* results correlate with the observed anti-tumor activity *in vivo* and suggest that the antitumor activity of the h1567 minibody in this CTCL murine model is mediated, at least in part, through Fcγ receptor IIIA (CD16A) engagement on mouse neutrophils to induce ADCC effector functions.

### Mechanism(s) of h1567 *in vivo* Tumor Killing in Human Peripheral Blood Mononuclear Cell (PBMC)-engrafted Mice Bearing Pre-established CCR4-positive Tumors

**Figure 3:**
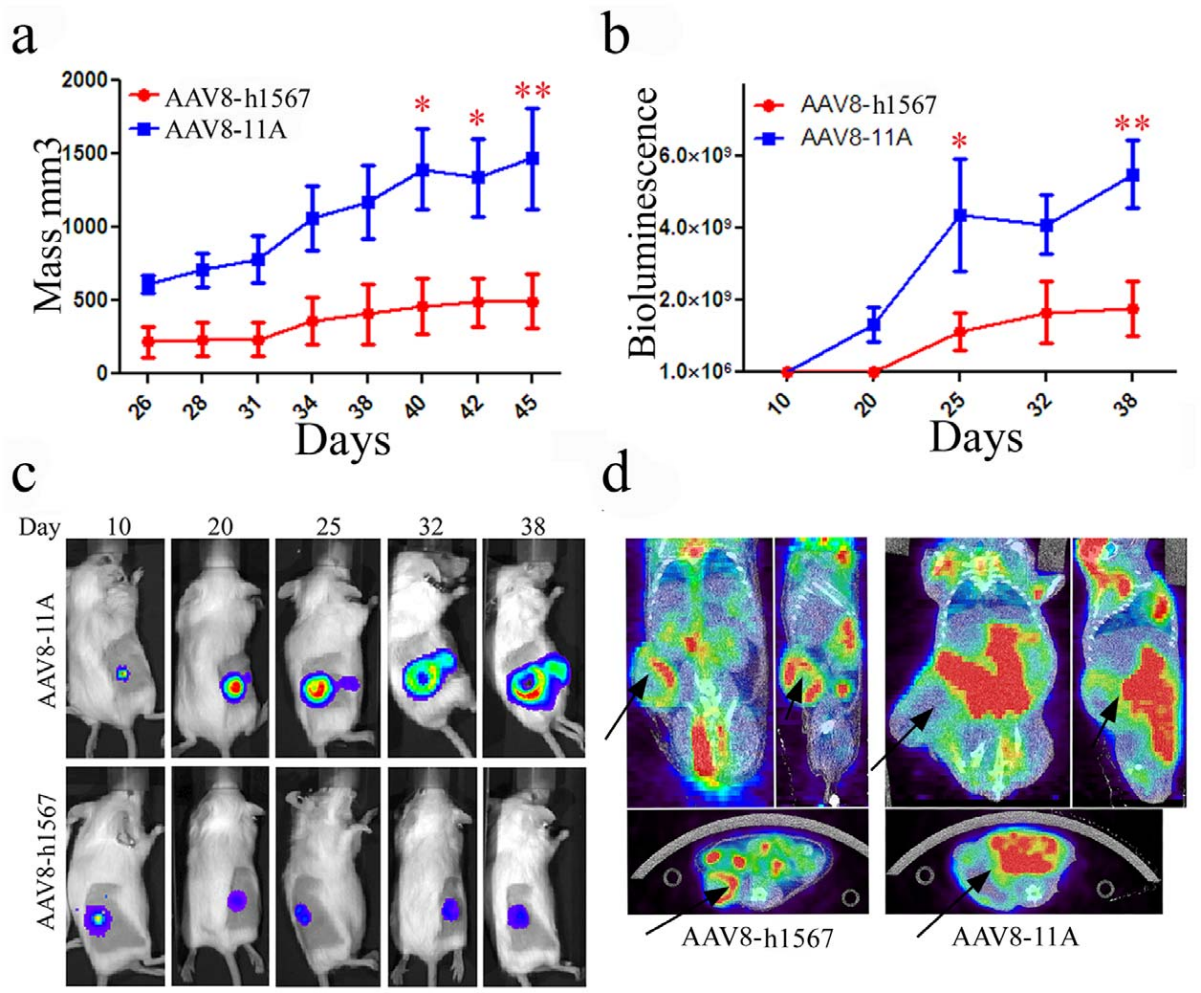
PBMC-mediated antitumor activity of the AAV8-derived h1567 minibody in a xenograft SCID-BEIGE mouse model. (**a**) Growth in tumor volume was quantified by caliper measurements. Tumor progression was significantly inhibited in the AAV8-h1567-treated group compared with the AAV8-11A control group. Mice were given a single intravenous injection of AAV vectors 11 days after inoculation of the tumor cells, which was followed by a single injection of PBMC on day 18. *P<0.05; **P<0.01. (**b**) Tumor growth was monitored *in vivo* by optical imaging and quantified weekly by bioluminescent imaging. *P<0.05; **P<0.01. (**c**) Sequential *in vivo* imaging of tumor growth over time in the tumor mouse model. Panels depict a representative mouse from each group. (**d**) Micro-CT/PET fusion images of representative mice 28 days after tumor inoculation. Representative coronal (left), sagittal (right), and transverse sections (below) are shown for both controls and treated mice. Arrows indicate tumor location. FDG PET revealed a decrease in glucose metabolism in AAV8-h1567-treated mice. Data shown are mean values ± SD.

The therapeutic CTCL model was further extended to evaluate the role of human effector cells in tumor cell killing using bioluminescence imaging (BLI) of luciferase expressing CCR4^+^ Mac-1 cells established by retroviral transduction. Ten SCID-BEIGE mice that were grafted with 1×10^6^ CCR4^+^ Mac-1 cells and developed equivalent sized tumors as detected on day 7 by BLI were divided into two groups. Eleven days after initial tumor cell inoculation, the AAV8-minibody vectors were administered intravenously. Next, human PBMCs (hPBMCs) were given by intraperitoneal injection 7 days after AAV vector administration. As shown in **Figure 3a**, treatment with AAV8-h1567 and hPBMCs resulted in substantial tumor growth inhibition compared to AAV8-11A plus hPBMC treated mice. Quantitative monitoring of tumor growth by *in vivo* BLI correlated with visible tumor growth, further confirming the tumor growth inhibition effect of AAV8-h1567 compared with control group (**Figure 3b**). A significant difference was observed between the control AAV8-11A and therapeutic AAV8-h1567 groups on days 40, 42, and 45 after tumor inoculation by caliper measurement and by days 25 and 38 by BLI (**Figure 3a and b**). Real-time whole-body BLI of a representative mouse showed that tumor growth was considerably inhibited in mice treated with AAV8-h1567 compared with control mice over the treatment period (**Figure 3c**). Analysis of micro-computed tomography/positron emission tomography (mCT/PET) images also revealed tumor growth inhibition with AAV8-h1567 treatment compared with the control group. While both AAV8-h1567 and AAV8-11A showed primary tumor growth 28 days after tumor inoculation, the tumor cells became much more locally invasive in the AAV8-11A treated group and showed increased metabolic activity as indicated by the accumulation of the PET tracer ^18^F-fluorodeoxyglucose (FDG) in whole-body images of mice (**Figure 3d**).

**Figure 4:**
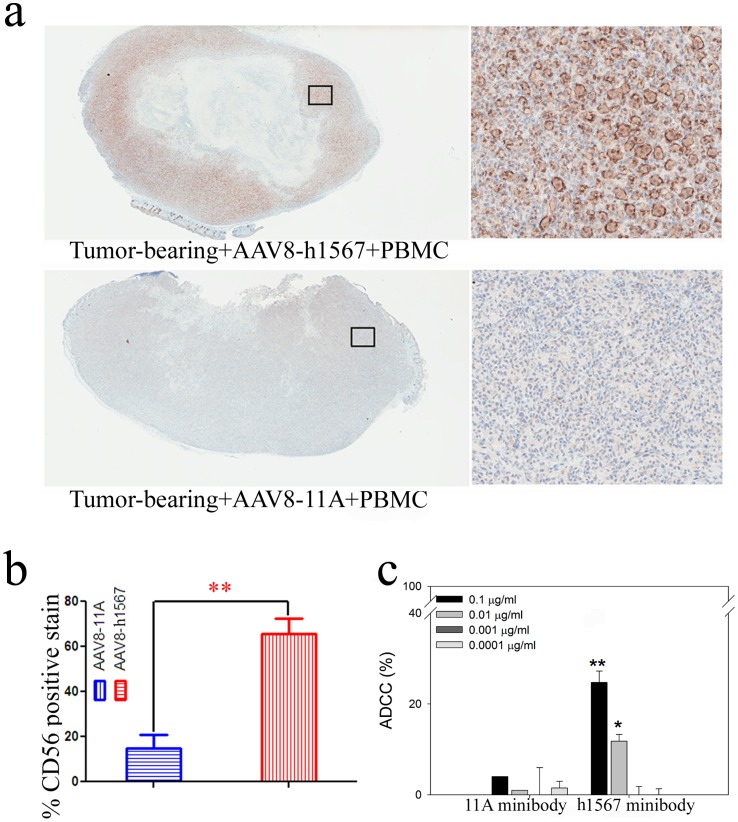
ADCC activity of h1567 minibody in a xenograft human PBMC-SCID/BEIGE mouse model. (**a**) Immunohistochemical staining of a representative tumor section with mAb directed against human NK cell surface marker CD56. The immunostaining shows highly positive CD56 tumor-infiltrating human NK cells (brown stain) in tumor from the SCID/BEIGE mice treated with AAV8-h1567 and hPBMCs (upper panel). Negative CD56 staining was seen in the tumor treated with control vector AAV8-11A plus hPBMCs (lower panel). Images are shown from whole tumor cut sections (left panels) and tumor sections at 20× magnifications (right panels). (**b**) The percentage of immunohistochemically detected tumor-infiltrating natural killer cells was plotted. A significantly higher percentage of tumor-infiltrating human CD56-positive cells were detected in the AAV8-h1567-treated mice group. **, p<0.01. (**c**) NK cell-mediated cytotoxicity was observed in a dose-dependent manner. Minibody concentrations from 0.0001 to 0.1 ug/ml were tested at an E:T ratio of 2∶1. The average and error bars (mean + SD) shown were calculated from triplicate wells of one experiment. The figures shown are representative of three independent experiments. *P<0.05, **P<0.01 when comparing h1567 minibody-treated and 11A control minibody-treated group. All data is shown as the mean ± SD.

To further assess the *in vivo* mechanisms of tumor cell killing in the AAV8-h1567 plus human PBMC treated group, the role of human NK cells, which also express FcγRIIIA receptors, was evaluated. In the AAV8-h1567 treatment group, a substantial increase in tumor-infiltrating human NK cells was observed, as shown by the intense CD56 immunostaining compared with control 11A treated mice (**Figure 4a**). Quantitative color deconvolution analysis showed a significantly increased staining in the mouse group treated with AAV8-h1567 compared with the control group treated with AAV8-11A (P<0.01; **Figure 4b**). Human NK cell-mediated ADCC activity was also evaluated *in vitro* using purified human NK cells as effector cells. As shown in **Figure 4c**, human NK cells were able to kill Mac-1 target cells in the presence of h1567 in a dose dependent fashion. Control 11A minibody showed only very low levels of killing. As both mouse neutrophils and human NK cells express FcγRIIIA receptors (CD16A) on their surface that can bind h1567, these *in vitro* and *in vivo* data strongly support that h1567 mediated killing occurs, at least in part, through FcγRIIIA engagement and activation of immune cell effector functions.

## Discussion

In this study, an AAV8-based therapeutic antibody gene transfer model was developed to evaluate a novel humanized anti-CCR4 monoclonal antibody h1567 as a therapeutic drug candidate against CTCL. The SCID-BEIGE mice that were used to establish this CTCL model lack T and B cells and functional NK cells [26]. High level, durable expression of the h1567 minibody was achieved after a single intravenous injection and significant anti-tumor activity against CCR4^+^ Mac-1 cells was seen in two animal treatment studies. These results provide the first *in vivo* evidence that mAb h1567 may be clinically active against CTCL cells and suggest that further studies should be undertaken to investigate its clinical efficacy.

Remarkable among the findings of this study is that a single intravenous tail vein treatment with AAV8-h1567 minibody resulted in a dose dependent increase in serum minibody levels that steadily increased over a two week period and remained at near peak levels through the end of this 15 week study (**Figure S1,** **Figure 1b**). The integrity of the minibodies was demonstrated biochemically *in vitro* and *in vivo* by several parameters including their CCR4^+^ binding activity, stable dimer formation and ease of recovery by protein A chromatography (**Figure 1** **c and 1d**). This scFvFc minibody format may be ideal for experimental AAV8 delivery since conventional mAb expression is derived from heavy and light chain genes, and it can be difficult to achieve the balanced expression of two genes within a single AAV vector that has a small packaging capacity (less than 5 kb), although a 2A self-processing peptide and furin cleavage have been successfully used to drive the expression of full-length rat IgG [27], [28]. For cancer immunotherapy, scFvFc minibodies appear to be promising because they have been shown to be functionally comparable with full-length IgG and have been successfully used to treat various tumors in preclinical studies [29], [30], [31]. As an added benefit, along with their bivalent antigen-binding avidity and intact antibody effector functions, they comprise a single polypeptide chain that does not require balanced heavy and light antibody chain heterocomplex associations and have a smaller molecular weight for better tissue penetration compared with whole IgG molecules [32].

The functional integrity of the h1567 minibody was also shown by its potent *in vivo* anti-tumor and *in vitro* killing activities. In the first treatment study (model 1), marked inhibition of Mac-1 tumor cell growth and increased survival was seen (**Figure 2a and b**) even though these SCID-BEIGE mice have profound immune cell defects including lack of T and B cells as well as impaired macrophage and NK cell effector functions [26]. Further IHC staining of the paraffin-embedded tumor tissues revealed a predominant infiltration of Ly-6G^+^ CD16A^+^ neutrophils only in the h1567 minibody but not control 11A minibody treated mice **(** **Figure 2c**). Furthermore, *in vitro* ADCC assay using purified mouse neutrophils as an effector cells demonstrated that h1567 induced significant lysis of CCR4^+^ Mac-1 cells, while no lysis was seen with 11A (**Figure 2e**). These results support the view that neutrophil-mediated ADCC is involved in anti-tumor activities following AAV8-h1567 gene delivery in the SCID-BEIGE CTCL mouse model.

The therapeutic SCID-BEIGE CTCL model was further extended to evaluate the role of human effector cells in tumor cell killing. In the second treatment study (model 2), AAV8-h1567 gene delivery together with human PBMCs was evaluated and a significant inhibition of CCR4^+^ Mac-1 tumor cell growth was again seen (**Figure 3**). The therapeutic effect of the h1567 was monitored using tumor size measurements and BLI. In comparison with the measurement of tumor volume, BLI analysis enabled earlier tumor detection and revealed extensive cell death in response to h1567 treatment (**Figure 3c**). The addition of microPET and CT images provided three-dimensional analysis of the primary tumor and further evaluation of the effectiveness of the AAV8-h1567 treatment *in vivo*. PET imaging indicated invasive tumor cell infiltration into surrounding tissues which was not seen in the h1567 treated mice (**Figure 3d**).

The FcγRIIIA receptor (CD16A) is the dominant FcγR involved in human NK cell-mediated ADCC. Treatment of CCR4^+^ Mac-1 tumor bearing mice with AAV8-h1567 and human PBMCs resulted in a marked increase in the number of tumor-infiltrating human CD56^+^ NK cells, suggesting that CD16A which is expressed on human NK cells is involved in this tumor cell killing through it’s interaction with the Fc portion of h1567, a finding that has been experimentally confirmed through Fc mutagenesis studies (data not shown). Moreover, *in vitro* ADCC studies with purified human NK cells demonstrated a concentration dependent killing by h1567 (**Figure 4c**). Thus the unifying observations from both treatment studies strongly suggest that the *in vivo* anti-tumor activity of h1567 is mediated, at least in large part, by ADCC through engagement of FcγRIIIA on mouse neutrophils (model 1) and human NK cells (model 2). Since Fc gamma receptors (FcγRs) of different types are present on a variety of effector cell populations, including NK cells, dendritic cells, macrophages, monocytes and neutrophils [33], [34], it is possible that FcγR engagement on other immune effector cells, not investigated in this study could also be involved.

MAb therapy for advanced CTCL has been proposed [3] and numerous trials with alemtuzumab (anti-CD52) have shown modest to moderate clinical effects [35], [36], [37]. A recent trial with low dose alemtuzumab has shown complete remission in 50% of patients with refractory leukemic forms of the disease and without infectious disease complications although it was found completely ineffective in the treatment of MF [6]. A mAb to CD4 (GenMab) has been designated an orphan drug for the treatment of MF by the FDA [38]. While both approaches are designed to eliminate CTCL cells, there can be significant adverse effects from either treatment. CD52 is expressed by virtually all T and B cells, and the elimination of all CD4-positive cells has well-known negative consequences [39]. Clonal malignant T cells in these CTCL patients express uniformly high levels of CCR4, but variable to low levels of other skin homing addressins, including CLA, CCR10 and CCR6. CCR7, which is also uniformly highly expressed on leukemic variants of CTCL with T_CM_ phenotype, is not expressed on the phenotypic T_EM_ cells that are found in MF skin lesions [7]. Thus, only CCR4 is uniformly expressed on all forms of CTCL and has a restricted expression pattern on normal T cells, including Tregs [40]. Indeed, a subset of malignant T cells in some CTCL have been shown to act as CCR4^+^ Tregs to suppress anti-tumor responses and may fuel disease progression [41]. A therapeutic mAb that could preferentially target all forms of the disease and reverse Treg mediated immune suppression would be a major advance in the effective therapy of CTCL. The activity of mAb1567 in abrogating Treg mediated suppression of T effector cell function is described elsewhere (DK. Chang et al., in press). MAb KM0761, is another humanized anti-CCR4 mAb that has shown promising results in CTCL animal studies [42] and in clinical trials for refractory Adult T-cell leukemia (ATLL) and peripheral T cell lymphoma (PLCL) where good clinical activity without severe adverse side effects was seen [43], [44]. Our data support further exploration of the clinical potential of therapeutic mAbs that target CCR4 in CTCL.

In summary, the results of the present study have validated the utility of an AAV8-based therapeutic minibody gene transfer platform for the rapid experimental evaluation of mAbs for the treatment of human cancer. Furthermore, this study showed that the AAV8-h1567 minibody inhibited the primary CCR4^+^ tumor burden, suppressed local metastasis and prolonged the survival time in tumor-bearing SCID-BEIGE mice. We remain hopeful that additional studies will support this humanized mAb1567 moving from bench to bedside.

## Materials and Methods

### Ethics Statement

All animal procedures were performed according to the recommendations in the Guide for the Care and Use of Laboratory Animals of the National Institutes of Health and in accordance with an approved protocol by the Institutional Animal Care and Use Committee of the Harvard Medical School (Permit Number: 04254).

### Cells

The human skin-tropic Anaplastic large-cell lymphoma (ALCL) cell line Mac-1, which was originally isolated in the laboratory of Marshall E. Kadin at Harvard Medical School [45], was cultured in RPMI medium supplemented with 10% fetal bovine serum (FBS), 0.06 mM 2-mercaptoethanol, and 500 µg/ml G418. Immunophenotyping of the Mac-1 cell line showed the expression of all known tumor-specific chemokine receptors, including high levels of CCR4, CCR7, and CXCR4. This MAC-1 cell line was stably transduced with a luciferase encoding retrovirus. HEK 293 cells were cultured in Dulbecco’s modified Eagle’s medium supplemented with 10% FBS and 1% penicillin/streptomycin (Invitrogen). All cells and cultures were maintained at 37°C in a 5% CO_2_ humidified incubator. Human PBMCs obtained from the Dana-Farber Blood Center were purified by a Ficoll-Hypaque density gradient centrifugation as described in the general protocol of Miltenyi Biotec Inc. (Auburn, CA). Mouse neutrophils were isolated from SCID-BEIGE mouse blood by Percoll density gradient centrifugation, as described [46]. Human NK cells were isolated from human PBMC using the NK cell isolation kit, according to the manufacturer’s protocol (Miltenyi Biotec, CA).

### Construction of AAV8 Vector Encoding anti-CCR4 Humanized scFvFc h1567 mAb

To construct the scFvFc h1567 minibody expression cassette, the scFvFc h1567 gene was PCR-amplified from a plasmid coding for the humanized anti-human CCR4 antibody that is derived from heavy and light antibody chains of mAb 1567 (R&D Systems, Inc) previously cloned in our laboratory (DK. Chang et al., in press) and inserted into the AAV-cloning vector pTRUF (obtained from the University of Iowa Viral Vector Core) at the restriction sites of Sfi1 and Not1. Consequently, to efficiently direct the expression and secretion of the single chain mAb, the pTRUF vector was modified by inserting the human IgG VH4 leader sequence and the Fc sequence (hinge, CH2 and CH3 domains) of the human IgG1 flanked by 145-bp and AAV2-inverted terminal repeats (ITRs) (**Figure 1a**).

### Viral Vector Production

Recombinant AAV8 viral vectors were produced using a helper virus-free system with some modifications [47]. Low-passage human HEK 293 cells were cotransfected by linear polyethylenimine (Polysciences) with three plasmids: the AAV cis-plasmid pTRUF encoding the human mAb gene expression cassette flanked with ITRs; the AAV-packaging plasmid p5e18 (2/8) containing AAV2 rep and AV8 cap genes; and the Ad helper plasmid pXX6-80 containing the VA RNA, E2, and E4 genes required for AAV propagation (obtained from Dr. Jim Wilson, University of Pennsylvania) [48]. At 48 h post-transfection, the cells were harvested, and the AAV virus extracted by freezing and thawing the cells. Subsequently, AAV was purified by two sequential iodixanol density gradients, concentrated, then desalted by centrifugation through Biomax 100-K filters (Millipore) according to the manufacturer’s instructions. Viral titers were determined as genome copy titers (vg), by quantitative real-time PCR using primers and probe speicific for AAV vector pTRUF [49]. Forward primer (5′-TCTGAGTAGGTGTCATTCTATTCTGGG-3′) is located at the end of the 3′-poly(A), and reverse primer (5′-CACTAGGGGTTCCTAGATCTCTCCC-3′) is at the beginning of the 3′ inverted terminal repeat (ITR). The probe (5′-TCTTCCCAATCCTCCCCCTTGCTGTC-3; FAM/TAMRA) is located in between.

Larger quantity of the AAV serotype 8 vectors encoding scFvFc 11A, control minibody specific for SARS [21], and scFvFc h1567 were produced at Harvard Gene Therapy Initiative (Harvard Institute of Medicine, Boston, MA) and used in the animal studies.

### Therapeutic Animal Models

SCID-BEIGE female mice aged 6–8 weeks were purchased from Charles River Laboratories and maintained in the animal facilities of Harvard Medical School. For therapeutic minibody gene transfer studies (Mouse model 1), mice were inoculated subcutaneously into the left flank using a 13-guage trocar with 2.5×10^6^ cells CCR4^+^ Mac-1 cells in 200 uL PBS. At one-week post-tumor inoculation, mice were injected intravenously through the tail vein in a single treatment of AAV8 vector encoding the anti-CCR4 h1567 minibody or the irrelevant control 11A minibody at a dose of 2×10^11^ v.g. (viral genomes) in 150 uL of PBS. For a human PBMC-engrafted mice model (Mouse model 2), mice were inoculated with 1×10^6^ luciferase-expressing CCR4^+^ Mac-1 cells. Eleven days after tumor cell inoculation, the tumor-bearing mice were injected intravenously via the tail vein with AAV8 vectors. Human PBMC were injected intravenously through a tail vein, to a final concentration of 1×10^6^ cells per mouse at 7 days post-AAV8 injection. Subcutaneous tumors were measured using calipers, and tumor volumes were recorded according to the formula V = ½×L×W^2^, where W is the smaller diameter and L is the larger diameter. Treated and control mice were euthanized when the tumor diameter reached 1.5 cm or when the mice were moribund. The mice underwent necropsy and the tumors were evaluated by histology and immunohistochemistry (IHC).

### Optical Imaging

Mice were monitored for tumor development and progression by both caliber measurement and Xenogen BLI. The latter was initiated for the monitoring of tumor growth 7 days after tumor implantation, which was repeated once a week. Mice were anesthetized with 3.5% isoflurane in an induction chamber, which was followed by the intraperitoneal administration of 50 mg/ml D-luciferin. For imaging, mice were maintained under 1.5% isoflurane anesthesia that was delivered through a nose cone. Whole body images were repeatedly acquired until the maximum peak of photon number was confirmed during various exposure times (10 s–1 min). Data were quantified using the time point that gave the highest photon number during the scanning time and analyzed using the Living Imaging software (Caliper Life Sciences, Hopkinton, MA).

### CT/PET Imaging

PET/CT scans were performed at the Harvard Medical School Imaging Core Facility. Mice were fasted for 12 h before the ^18^F-FDG injections, but provided water *ad libitum*. For ^18^F-FDG injection and imaging, mice were anesthetized using 2% isoflurane. The animals were then intraperitoneally injected with 7.4 MBq (200 µCi) of ^18^F-FDG, allowed to regain consciousness, and then kept at 37°C until imaging. Imaging was started 30 min after the intraperitoneal injection. Mice were imaged in a chamber that minimized positioning errors between PET and CT to less than 1 mm. Image acquisition time was 10 min. Images were analyzed using AMIDE software [50]. All regions of interest were defined on fused PET/CT images to ensure reproducible positioning.

### Protein Expression and Purification

HEK 293T cells (ATCC, Manassas, VA) were transfected with the AAV-coding plasmid containing the minibody-expressing constructs using Lipofectamine 2000 (Invitrogen, Carlsbad, CA). Three days after transfection, the minibodies were purified from the supernatants with protein A sepharose affinity chromatography. The *in vivo* production of AAV8-minibodies was generated by i.v. injections into SCID-BEIGE mice as described above. Levels of minibodies in the serum were measured in duplicate using a human IgG ELISA quantitation kit according to the manufacturer’s protocol (Bethyl Laboratories, Inc., Montgomery, TX).

### Western Blot Analysis

Western immunoblotting was performed on protein A column purified samples containing *in vitro* synthesized minibodies and *in vivo* AAV8-derived minibodies. The proteins were separated by SDS-PAGE under reducing or nonreducing conditions and electrophoretically transferred onto a nitrocellulose membrane using the iBLot dry blotting system (Invitrogen). After blocking with 5% skim milk overnight, the blot was probed with an AP-conjugated human IgG-Fc antibody that was diluted 1∶30,000 in blocking buffer for 1 h at room temperature. Excess conjugate was removed by five washes with Phosphate buffered saline containing 0.1% Tween 20 (PBS-T). The detection of protein was performed by incubating the membrane with BCIP/NBT alkaline phosphatase substrate (KPL).

### Flow Cytometry Analysis

The biological activity of the *in vivo* AAV8-derived h1567 minbodies was analyzed by fluorescence-activated cell sorting (FACS) for binding activity. Mac-1 cells or 293T-CCR4 cells were washed with PBS supplemented with 0.5% bovine serum albumin (PBS-B) and then incubated with *in vivo* produced h1567 for 1 h at room temperature, which was followed by incubation with anti-human IgG-Fc conjugated to fluorescein isothiocyanate (FITC). Flow cytometric analysis was performed using BD FacsCalibur (BD Biosciences, San Jose, CA) and FlowJo data analysis software (Tree Star, Inc., Ashland, OR).

### Immunohistochemistry and Quantification of Cell Staining

Immunohistochemical staining was performed at DFCI/Harvard Cancer Center Research Pathology Core. For qualitative and quantitative immunohistochemical analysis, formalin-fixed and paraffin-embedded tissue sections were stained with antibodies directed against Ly-6G on the surface of mouse neutrophils and human CD56 antigen on human NK cells. The stained slides were then scanned using the Aperio ImageScope (Aperio Technologies, Inc., Vista, CA), and full tumor sections were selected for quantitative analyses. The percentage of positively stained cells in the entire tumor sections was calculated using a color deconvolution algorithm.

### In vitro Antibody-dependent Cell Cytotoxicity Assay

ADCC was performed using the lactate dehydrogenase (LDH) release assay method, according to the CytoTox96 non-radioactive cytotoxicity assay procedure specified by the manufacturer (Promega, Madison, WI). Mouse neutrophils purified from SCID-BEIGE mouse or purified human NK cells from PBMC was used as effector cells and CCR4+ Mac1 tumor cells were used as target cells. Briefly, purified SCID-BEIGE mouse neutrophils or NK cells were plated at a density of 1×10^4^ cells per well in a round-bottom 96-well plate in the presence of h1567 or 11A minibodies. After 1 h of incubation, freshly prepared effector cells were added at an effector-target cell ratio (E:T) of 80∶1 (mouse neutrophils) or 2∶1 (human NK cells). After 2 h incubation at 37°C, supernatants of each well were recovered by centrifugation at 300×g for 5 min. LDH activity in the supernatant was determined by measuring absorbance at a wavelength of 490 nm. The cytotoxicity (%) was calculated according to the following formula:
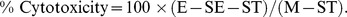
where E is the LDH release by effector-target coculture, SE the spontaneous release of the LDH from the effector cells, ST the spontaneous release of the LDH from the target cells and M the maximum release of the LDH from the target cells incubated with lysis solution (10% Triton-X). All measurements were done in triplicate.

### Statistical Analysis

Statistical analyses were performed using 2-way ANOVA with Bonferroni post hoc tests and unpaired 2-tailed t-tests using GraphPad Prism 5 (GraphPad Software, Inc., La Jolla, CA). P values less than 0.05 were considered statistically significant.

## Supporting Information

Figure S1 **Dose dependent expression of h1567 anti-CCR4 minibody.** Nude mice (4 mice per group) were treated one time by tail vein injection with AAV8-h1567 viral vectors at the two concentrations shows. PBS buffer treated mice served as controls. Mice were bled at the indicated time points over 15 weeks and their h1567 scFv-Fc levels were determined by ELISA on anti-human Ig capture and detection.
(TIF)
