# Supplementary figures and images for: Human Anti-CCR4 Minibody Gene Transfer for the Treatment of Cutaneous T-Cell Lymphoma
Source: PLoS One. 2012 Sep 4;7(9):e44455. doi: 10.1371/journal.pone.0044455 (PMC3433438; doi:10.1371/journal.pone.0044455)

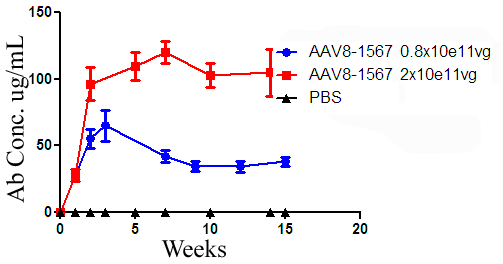

Supplement: Figure S1 — Dose dependent expression of h1567 anti-CCR4 minibody. Nude mice (4 mice per group) were treated one time by tail vein injection with AAV8-h1567 viral vectors at the two concentrations shows. PBS buffer treated mice served as controls. Mice were bled at the indicated time points over 15 weeks and their h1567 scFv-Fc levels were determined by ELISA on anti-human Ig capture and detection. (TIF) [file pone.0044455.s001.tif]
